# How External Environment and Altruistic Traits Drive Eco-Centric Entrepreneurial Intention Among Youth in the Post-COVID-19 Era?

**DOI:** 10.3389/fpsyg.2022.817619

**Published:** 2022-05-03

**Authors:** Yudong Weng, Ahmad Tisman Pasha, Muhammad Shaukat Malik, Muhammad Umar Farooq, Siraj Hussain

**Affiliations:** ^1^School of Economics, Jilin University, Changchun, China; ^2^Institute of Banking & Finance, Bahauddin Zakariya University, Multan, Pakistan; ^3^Department of Sociology, Bahauddin Zakariya University, Multan, Pakistan

**Keywords:** green entrepreneurial intention, warm glow, market orientation, normative support, regulative support, post-COVID-19 era

## Abstract

Green entrepreneurship is a recent business phenomenon that is related to ecological issues. In the time of COVID-19, every business entity is looking for a unique way to be more resilient and noticeable. In this regard, green entrepreneurs hold the potential to manage scarce resources, fulfill social responsibility, and put forward the solution to environmental degradation in the new normal of the post-COVID-19 era. The current venture investigated the opportunity recognition and readiness behavior to execute green entrepreneurship intentions during the post-COVID-19 situation (specifically by underlining the role of market orientation). The present model examined the institutional theory. It put forward the opportunity recognition behavior in the post-COVID-19 era, which can improve youth readiness to opt for green entrepreneurship. The study collected data from 196 university graduate students *via* online sources by using purposive sampling. The study found that the proposed hypothesis has been proved significant while defining green entrepreneurial intentions. Particularly, the study showed that warm glow was the strongest factor to predict green entrepreneur intention. Moreover, this study can contribute to employing the institutional theory as a novel aspect in the academic sphere.

## Introduction

Green entrepreneurship regards social or environmental problems. It is a combination of entrepreneurship and the environment. Green entrepreneurship has sought solutions to various social and environmental problems through innovative ideas. This concept has entered the market by swapping traditional products. Green entrepreneurship lays foundations on renewable energy and pro-environmental and green principles (Gast et al., 2017). Green entrepreneurs trigger the exploration of green goods and mechanization within the market. Green entrepreneurship can protect and enhance the natural environment if it is materialized into collective action by the masses (Lotfi et al., 2018).

Green entrepreneur intention (GEI) is an individual’s perception of certain actions whether they are significant to influence the environment or not. People are sensitized to green innovation when they have to foster GEI in themselves. Cheung and Lee (2009) demarcated between the two sorts of green intentions, the first is continuance intention, which refers to the user or the consumer’s decision to consume or use a particular service or the products they are utilizing earlier. It leads to long-term sustainable effects. While the second one is recommendation intention conveyed to the consumer by someone other than the organization. Correspondingly, the entrepreneur intention is captioned as the capacity to run one’s own business or gaining self-employment, it is based on the creative venture that spurs the era of innovation in the traditional markets (Fitzsimmons and Douglas, 2011).

The aim of the study in the underlined model is based on how the unique institutional factors lead to green entrepreneurial intentions among youth. In addition to this, the exogenous factors are normative support, regulatory support, and the relevant knowledge experience that trigger the behavior regarding the green entrepreneur’s intentions. However, warm glow and market orientation will operate as mediators within the model; both mediators are the altruistic traits of the individual that will predict green entrepreneurship intentions. Lastly, the green entrepreneur’s readiness is the cognitive factor that has been chosen as the moderator of the study. Overall, the model will elucidate how young entrepreneurs adopt green entrepreneurship.

Nonetheless, the theoretical and practical implications of the current research may provide a framework for academicians and policymakers to increase green entrepreneurship regarding certain conditions. This will also add a target for organizations to meet in the contemporary requirements of pro-environmental behavior. This will increase institutional performance by harnessing the potential of youngsters in the new normal of the post COVID-19 era. The present study focuses on the performance of the institutional forces by measuring green entrepreneurship. The concerned institutional forces are related knowledge and experience and regulatory and normative support. The study contributes uniquely by investigating green entrepreneur readiness as the moderator in this mode. At the same time, its significant role in the academic spectrum increases by adding altruistic values as a mediator for the first time in this framed model of the study.

To address the research gap, the current study examines the influence of the external environment to implement green entrepreneurial intention among youth. We create our research model from institutional theory. Specifically, we considered the factors of the external environment (i.e., normative support, regulative support, related knowledge, and experience) and inspect their impact on green entrepreneurial intention. We use two mediating variables (market orientation and warm glow) and one moderating variable (green entrepreneurial readiness). This study presents three important contributions that have never been discussed before in the aspect of green entrepreneurial intention. First, we investigate the role of institutional forces during the survey of green entrepreneurship. These institutional forces are normative support, regulative support, and related knowledge and experience. Second, we use green entrepreneurial readiness as a moderator for the first time. Finally, the mediating role of altruistic value (warm glow) has not yet been studied in the case of green entrepreneurship. In other words, the current study extends the work of Barba-Sánchez et al. (2022) as the current research takes more dynamic aspects other than social norms, perceived behavioral control, and attitude into consideration.

## Literature Review

### Green Entrepreneurship and Its Readiness

Shah and Soomro (2017) remarked that young individuals engage in entrepreneurial activities by initiating a Miner scale business. Contrarily the aspect of green entrepreneurship has been ignored while studying the dynamics of entrepreneurship. The role of green entrepreneurship is vital in starting any entrepreneur activity (Soomro et al., 2020). University education plays a vital role in the entrepreneurial talents of youngsters (Tognazzo et al., 2017). Accordingly, coaxed sustainability is a means through which long-term effects can be anticipated (Sterling, 2010). The entrepreneurial spirit increases the tendency to build green corporations with effective measures (Hockerts and Wüstenhagen, 2010). The quest to promote green entrepreneurship can be seen at the individual and the global level.

Consequently, motivation and passion derive green behavioral awareness among individuals (Masurel, 2007). Similarly, green entrepreneurship requires long-term initiatives that may be executed through delicate steps; in contrast, green intentions may be fostered initially, but their long-term impact can be seen over a considerable time (Yi, 2020). The massive steps needed to increase green entrepreneurship can create a new target and promote behavioral patterns to spur on long-lasting entrepreneurship activities (Wei and Nordin, 2020). Intention has been recognized as one of the basic factors to outline business contingencies and eventually influence them, and access concerning inspecting it, specifically from a green aspect (Yasir et al., 2021).

Green entrepreneurial readiness is the preparedness to execute something environmentally friendly. Green entrepreneurial readiness prepares individuals to choose conservation and sustainability within business models. They are deliberated to decrease the negative impacts of their innovative ventures to protect the environment (Gibbs and Neill, 2012). For this commitment, they may create an alliance with philanthropists and local partners by taking green initiatives. They are aimed to enhance green products, such kinds of products are made with sustainable procedures and materials that do not harm the environment (Hsu et al., 2017). Green entrepreneurship emerges within the economic problems scenario and puts forward the solution to environmental issues; they address environmental anxieties and forge social responsibility. Various Western regimes have promoted green entrepreneurship as it resolves economic issues and promotes a sustainable societal approach (Hua and Wang, 2019). Green entrepreneurship has been hailed by society, and this has changed the magnitude of social actions into readiness (Wang et al., 2019). The level of consumers’ awareness regarding the problems created by certain products generates anxiety, and they start to use green products giving rise to the demand for green entrepreneurial activities (Nikolaou et al., 2011). This creates attitudes that help to promote eco-centric behavior. There has been much concentration on green entrepreneurial activities (Arli et al., 2018). Besides this, there is a need to rethink the institutional environment with the help of organizations and business entities. The increased support of green entrepreneurship from different stakeholders of society can lead to a just and balanced environment, and youth’s intentions can be aligned with green intentions (Ahmed et al., 2021). The cause of containing a commitment to the environment is that the younger generation is close to sustainable values. It has been displayed that the ability-based aspect provides against pupils’ green entrepreneurial sense (Hameed et al., 2021).

### Market Orientation and Warm Glow

Market orientation is a business approach to business that prefers to categorize the customers’ requirements and desires and satisfy them through specific products and services (Narver and Slater, 1990). Market-orientated companies always focus on the needs and desires of their targeted markets and populations. This becomes the critical component of their research and development for their upcoming ventures. On the contrary, the traditional markets focus on strategies to increase their selling points rather than fulfilling the required needs of the consumers. Accordingly, market orientation develops strategies that point out how the consumers’ needs are changing (McKelvie and Davidsson, 2009). They follow up the feedback and consider it significant to bring changes in their respective products. Consequently, their sale and product services increase. It is also involved in the long-range decision-making of the companies. They are always updated with the customer’s views and meet their concerns. Perhaps research plays an essential role in market orientation (Astuti et al., 2021). Market orientation is an obscured resource that increases management awareness regarding consumer wants for green products (Habib et al., 2021). Data analysis has an important role to probe the behavior and the trends of the customers. Sufficient knowledge of the product can anticipate the future consumption of that particular product. Consequently, companies alternate the characteristics of their products from time to time. Perhaps companies with innovative and adaptive behavior understand market dynamics and outperform their competitors.

Warm glow theory focuses on the emotional outcomes of helping other people. Individuals feel pleasure when they help others. The warm glow was theorized by James Andreoni, who stated that people are overwhelmed with satisfactory feelings without seeing the actual effects of their actions by helping others (Taufik et al., 2015). In warm glow, people have both egoistic and altruistic characteristics. Egoism is derived from their selfish motivations (Bouman and Steg, 2019). The rationale behind this can be the credit that people receive from their good deeds. However, here, the altruistic tendency can be bifurcated between the imperfect altruist and the pure altruist; the former’s behavior is derived from the joy of giving to others while the latter focuses on the sentiments to assist others. Interestingly warm glow is originated without any financial rewards. Warm glow is a significant contributor to public interests’ greater good and collective actions (de Groot and Steg, 2008). Warm glow has been examined by sociology, management studies, and the environmental and health care disciplines. It has been observed that warm glow is resilient across various cultural contexts. Warm glow has a vital role in an entrepreneur’s activities (Hartmann et al., 2017). It gives them the motivation to deliver for the people. Language contributes as an essential element in the entrepreneurial choice because it builds the narratives that catch the attention of different people (Muntoro et al., 2020). In another sense, the concept of warm glow enhances strike intentions over belief (Bhutto et al., 2021). Green warm glow can spur pro-environmental actions in society.

### Normative and Regulative Support

Norms set forth the parameters concerning entrepreneurial patterns. People with a background of strong norms can create influential outcomes in their entrepreneurial ventures (Seelos et al., 2011). Therefore, normative support can be an important element of entrepreneurial behavior, but the research has gaps in examining the impact of social norms and certain actions of entrepreneurship (Seelos et al., 2011). The influence of norms and their distinctions from values and other cultural concepts can be distinguished from the Auguste Comte and Durkheim theoretical approaches. Social norms are transmitted by guilt, shame, and formal social sanctions that motivate individuals to avoid their selfish interests and priorities for the collective benefit of society (Turulja et al., 2020). It is the power of social norms that they approve or disapprove all the actions in the form of social reactions, i.e., family, friends, coworkers; perhaps this sort of social pressure can be a key driver of entrepreneurial actions to deliver and work for green behavior (Valdez and Richardson, 2013) as the social norms possess the capacity to provide the efficiency of social appreciation so the individuals focus on pro-environmental action while creating, evolving, and executing the idea of the entrepreneurship (Doanh et al., 2021). When entrepreneurs can suggest the market and eventually create suitable norms, they will produce the latest aspects for entrepreneurship and attain notable importance for it, impacting the surrounding quality (He et al., 2020). Authors argue that the new normal in the post-COVID-19 era demands psychological support where the normative environment for entrepreneurs can help to improve the economic stability and growth of developing nations’ economies.

Regulatory support refers to the policies and the legislation by different regimes about certain phenomena. Regulatory support can be a vital factor to foster green entrepreneurship in any cultural and social setting (Seelos et al., 2011). The regulatory supports set forth the footprints of the market structure from which one can harness business potential. Regulatory stability can produce a balanced environment for entrepreneurial activities (Valdez and Richardson, 2013). The rule of law, writ of government, strict security, and property rights are the basis where green entrepreneurship is fostered and developed (Urban and Kujinga, 2017). The improved regulatory status catches innovative ideas and strengthens marginalized groups, motivates entrepreneurs, and fortifies the ecosystem governance system (Eckhardt and Shane, 2003). This can lead to the climax of entrepreneurship and relevant activities ultimately adding to the pro-environmental steps (Verheul et al., 2000). But its ramifications are subject to long-term initiation and the sustainability of the innovative green ideas. The regulatory indicators provide the relationship with the self-efficacy of the entrepreneurs (Fuentelsaz et al., 2019). The appreciation of the regimes and the penalties can lead to better innovative green entrepreneurship. The government’s taxes, loans, and the various conditional finance schemes can also pose better and positive effects on green entrepreneurship (Mohd Noor et al., 2021). The government base for green entrepreneurship assigns a more renewable environment and may be a basic effort against a wider environment-friendly association and preservation of reserves for the approaching era (Alwakid et al., 2020). It is critically essential in the post-COVID-19 era, as small and medium business entities are less resilient, agile, and adaptive due to restrictive economic growth.

### Related Knowledge and Experience

The role of related knowledge experience lays stress over innovative ventures and promotes the competitive environment and decisions of the youth (Lenox and King, 2004). The related knowledge experience escalates business activities and puts forward new thoughts and creativity. It also apprehends the opportunities and market trends and helps to launch new features demanded by customers concerning long-lasting effects (Shinnar et al., 2018). It has been revealed that related knowledge can lead to the sustainability of the environment and amendment enterprise (Bolisani and Bratianu, 2018). It observes the external threats and prepares for the resilience of launched green entrepreneurs. The engrained literature suggests that there is an obvious contribution of skills and related knowledge experience in any of the green entrepreneurship activities. On the other hand, the lack of knowledge experience seems to be lethal for successful ventures because it increases the tendency of marginal risks. The sole knowledge experience has adequate efficiency as it can lead to the climax of entrepreneurship. The outcome of knowledge can yield the best outcomes with ability, creative thinking, and valuable previous experience. Knowledge experience and green innovation have been closely linked with each other. This creates the basis of novelty and green entrepreneurship (Ha and Kurczewska, 2019). Experience in entrepreneurship has alone concisely become a successor to what knowledge is derived from experience. Experience has dual aspects and it bears what is happening in real time as described by entrepreneurship (Ha and Kurczewska, 2019).

## Theoretical Framework and Hypotheses Building

The institutional theory puts forward the resilient and profound features of the social structures. It takes into account the particular procedures through which structures, rules, schemes, routines, and norms function as an authoritative parameter for human societal behavior (Eckhardt and Shane, 2003). This concerned theory elaborates on how these factors emerged, flowed, adopted, and adapted regarding time and space and how these elements fail to deliver and disuse and decline. There are two versions of the theory, the old and the new one. Institutional theory is categorized into the three basic elements that set forth its basis, they are norms, rules, and routines; perhaps societal norms are coercive in any society, either on the personal level or the social level. The ideas originated from these certain norms, values, and routines ascertain the reality and produce the knowledge of an individual.

*Norms* are the standard pattern of the behavior that formulate the individual opinion regarding certain things. Smallbone and Welter (2012) believe that norms maintain control over the individual and they produce the thoughts and sense-making within particular communities, although the cultures configure these norms depending upon the individual’s potential to cognize these norms. Norms distinguish between the desired and the undesired paths of society, while cultural cognitive norms are the perceptions derived from the norms by an individual. Affirmation from the reactions proves that the common norms do not attribute high worth to entrepreneurial exercises (Ogunsade et al., 2021). Nevertheless, in the present study, normative support has been adopted as an independent variable to seek out the favorable or non-favorable situations concerning green entrepreneurs. In the present theoretical foundation, norms have been adapted as normative support within the mapped-out model. In addition to this, the other aspect of the institutional theory is rules, they have been adapted as the regulatory support within the present contexts. While lastly routines have been made the basis in terms of knowledge experiences to measure green entrepreneurship intentions.

*Rules* are captioned as the regulative mechanisms of society that are present in the form of laws. Scott considers that rules include and anticipate the range of individual actions within a legal circumference; they keep checks over the patterns of the society, forge the concept of compliance, and put forward the penalties and rewards to control and predict the individual’s actions. Formal institutions are considered as accurate, arranged, and authorized rules (Bremer et al., 2021).

*Routines* refer to the regular actions accomplished by the individual during a particular period. Routine is an essential constituent element of institutional theory. They set forth the basis of knowledge and information by the unique experiences of the individual. Accordingly, the routines foster and polish individual skills when they have repeated certain activities regularly. Somehow it makes an individual an expert within the specific field. Knowledge, ideas, and skills have a significant role in the development and achievement of any organization’s projects (Drucker, 1995). The warm glow theory has its basis in the emotional outcomes of doing good for others. It illustrates that masses encounter satisfaction while helping others. This theory revolves around the personal gains that are based on the emotional satisfaction of the individuals. It is based on the selfish pleasure resulting from the individual’s behavior.

The success of any venture relies on the knowledge extracted from routine activities and comes up in the shape of experience or the skill of the individual (Carlsson et al., 2013). Based on the aforementioned discussion, knowledge experience has been laid out based on routines. Routines additionally mention diverse things. Scholars have discussed and noticed routines at various aspects of search, and, with altered phrasing, have been annoyed by what routines require (Wolthuis and Hubers, 2021). Smallbone and Welter (2012) suggest that norms are the strong societal and cultural patterns that lay pressure over individuals and put them in a situation to confirm the existing cultural patterns. Norms are codes that configure the social behavior of individuals. Norms are derived from cultural cognitive segments and they are kept in check over individuals. Nonetheless, norms that have been engrained in any culture support green entrepreneurship because they strengthens the collective welfare of society. The other hypothesis of the examined study is a market orientation that can be captioned as the strategies to change the appraisals keeping in view the customer needs and the demands. It creates a better opportunity for the business (Narver and Slater, 1990). Perhaps market orientation can spur greater opportunities for entrepreneurs to excel and grow accordingly.

The behavior and intentions of individuals are determined by normative support. This normative support is influenced by the social circle of the individuals including family, colleagues, and the neighborhood. Normative support can be captioned as norms penetrating the individual’s life creating restrictions for certain things and gratifying particular social values. It has been elucidated by Wang et al. that individual willingness to adopt green behavior increases when influenced by normative support. Market orientation refers to the valuable and rare resource of the organization to foster differentiation and customer value to increase competitive edge (Green et al., 2015). In addition to this, normative support enhances market orientation owing to societal pressures which yield green entrepreneurship.

The rationale behind the above-drawn hypothesis of normative support and market orientation can be illustrated here. Ye et al. (2020) put forward normative support as a moderator to seek the reasons why people develop green intentions. Accordingly, Jancenelle et al. (2017) argued and reflected on the relationship between market orientation and subsequently with normative support and pro-environmental behavior. Similarly, it has been found that normative support can foster green initiatives in society (Wang et al., 2019). Nevertheless, both normative support and green intentions can predict preferences for green entrepreneurship by youth.

Regulatory support can be an important factor to promote green entrepreneurship because it indicates the support of the government and legal implantations regarding certain things. Valdez and Richardson (2013) believe that significant measures and regulatory support with strong financial policies can create a suitable environment for green entrepreneurship. The regulatory support to punish people and appreciate people can induce collective actions to instill green intentions (Urban and Kujinga, 2017). Consequently, green entrepreneurship can exert positive effects on the institutional status (Estrin et al., 2013). The second hypothesis has been framed by keeping in view two studies, firstly Williams and Spielmann (2019) who carved out the impact of regulatory support on small and medium enterprises (SMEs) and the concerned market orientation. Correspondingly, Ye et al. (2020) articulated that regulatory support can influence the intentions to utilize green products. Regulatory support is the significant factor that correlates with the intentions to adopt green entrepreneurship. The role of the statutory bodies and the flexibility of the regulations of the market persuade the masses toward the adoption of behaviors. In addition to this, governments always foster an environment in terms of market orientation, as market orientation becomes a significant factor to motivate people into choosing green entrepreneurship.

The second hypothesis of the current venture will derive how regulatory support can be effective for market orientation among youth who want to execute green entrepreneurship. However, Dutta et al. (2015) put forward that the relevant knowledge of entrepreneurs yields the intention toward green activities. It is the role of entrepreneurs that can take market orientations to the forefront. Entrepreneurs can comprehend market changes, owing to their abundant knowledge and experience (Ye et al., 2020). On the contrary, insufficient knowledge and concerned experience can create impediments to initiating green entrepreneurship (Chan et al., 2018). Nevertheless, the hypothesis was framed to seek out the impact of knowledge and related experience over market orientation among green entrepreneurs.

H1 (a–c): Normative support, regulatory support, and knowledge/experience positively influence the perceived market orientation (MO) among (potential) green entrepreneurs.

Green actions are subject to the normative systems of society that configure human behavior to act on desired or undesired patterns of society. The mechanism of norms is systematic as they appreciate good deeds and prefer to punish wrongdoings as per the intensity of the actions (Davis et al., 2018). Perhaps warm glow can accelerate the actions of the individuals, but they are also a consequence of impure norms (Van Der Linden, 2018). Somehow the warm glow rationale is viewed alongside the narratives of green attitude (Hartmann et al., 2017).

In addition to this, strong regulatory support can foster an era of opportunities for entrepreneurship. The procedures of regulations can increase the chances of engagement in green activities; the easy process of attaining a license, reduced taxation, comprehensive financial schemes, and smaller interest rates can be the triggering factor in entrepreneurial activities. Along with all this, the significance and vitality of creative thoughts cannot be ignored. The strength of ideas and directions can streamline activities concerning laws and regulations. The enterprise has to be confined within this circumference because being environmentally friendly will bring in business (Zulfiqar et al., 2019b). Regulations that are strict and promote environmental concerns can create an ecological balance (Zhang et al., 2018). The role of governmental and non-governmental agencies can create better outcomes in this regard. Hörisch et al. (2014) depicted that related knowledge experience can have positive and long-lasting impacts irrespective of SMEs. Wang et al. (2013) argued that skills and knowledge experience establish the basis of the green environment and a balanced society. Contrarily related knowledge experience also impacts the environment and the relevant natural ecology while affecting green intentions (Taufique et al., 2017).

H2 (a–c): Normative support, regulatory support, and knowledge/experience positively influence warm glow (WG) among (potential) green entrepreneurs.

Market orientation can create maximum benefit and sustainability. Market orientations entail the holistic pictorial view of business markets. The comprehension of market orientation is the indicator to penetrate the market because, through this, one understands the market values, existing product flaws, and the systems that can be advantaged later on. By evaluating and adapting to the needs of the consumers, one can create new dimensions of the business (Suliyanto and Rahab, 2012). The liaison and the cooperation between the organizations can create a suitable enterprise for business. This motivates the companies to launch new and creative goods with innovative mechanisms in the markets (Del Carmen Martinez Serna et al., 2013). Market orientation can enhance the invention and the creative trends in terms of the services industry and corporations (Altuntaş et al., 2013).

Liñán (2008) remarked that certain norms increase entrepreneurial intentions. Human norms and ethics are key contributors to pro-environmental behavior (Hartmann et al., 2017). Human values and standard patterns can have better ramifications for certain behaviors (Chen et al., 2018). Donaldson (2019) remarked that specific behavior and commitment to the welfare of society can yield green entrepreneurial activity. Thus, based on the aforementioned argument the following statement has been hypothesized:

H3 and H4: Market orientation (MO) and altruistic warm glow (WG) positively influence green entrepreneurial intentions.

Despite keen observation by researchers to investigate green entrepreneurial intentions in the academic spectrum, only a few of studies have paid attention to particular variables like market orientation and readiness regarding green entrepreneurship (Sahoo and Panda, 2019). The advantages of green entrepreneurs will increase the individual ability to encounter different hazards in business. This enhances the individual capacity to encounter the challenges and meet the market needs (Zulfiqar et al., 2019a). Based on the above two studies, the researchers have framed the hypothesis to seek out the moderating impact of green entrepreneurial readiness on market orientation and the green entrepreneurial intentions among youth. The need for green entrepreneurship is obvious, but only those individuals who can follow social norms and keep priorities focused on the natural concerns of the environment show inclination (Wang et al., 2013). The following hypothesis can be proposed:

H5 (a and b): Green entrepreneurial readiness (GER) significantly moderates the relationship of market orientation (MO) and warm glow (WG) with green entrepreneurial intentions (GEIs).

## Research Methodology

### Measurement

The researcher opted for authentic and reliable scales to measure green entrepreneur intentions and the other proposed constructs in the study. Specifically, the measured items for regulative and normative support were adapted from Urban and Kujinga (2017). Market orientation was measured by adapting the research of Leal-Rodríguez et al. (2018). Green entrepreneur readiness was measured by adapting Natrajan’s (2019) work. Besides this, the construct to map entrepreneurial intentions and warm glow was adapted from the work by Ye et al. (2020). Instrument items for each construct are listed in Table 1. The five-point Likert scale was employed where 1 was marked as highly disagree and 5 as highly agree. In addition to this, the validity and reliability of the constructs were prioritized before initiating the proper data collection through the pilot study. To accomplish the pilot study, data were collected from 13 students. Moreover, the instrument was revised as per the recommendations of the participants in the pilot test.

**TABLE 1:** Adapted measurement of the constructs.

Constructs	Items	
Normative support (NS)	NS1	Turning new ideas into initiatives is admired in this country
	NS2	In this country, innovative and creative thinking is viewed as a route to success
	NS3	Entrepreneurs are admired in this country
Regulative support (RS)	RS1	Government organizations assist individuals in starting their initiatives/ventures
	RS2	Local and national governments support individuals who are starting an initiative/venture
	RS3	The government sponsors organizations that help new initiatives/ventures
Market orientation (MO)	MO1	We regularly analyze and track the needs of our youth
	MO2	Objectives are determined by our youth satisfaction
	MO3	The strategy to obtain competitive advantage is based on the understanding of our youth’s needs
	MO4	We regularly measured our youth’s satisfaction
Knowledge and experience (KnE)	KnE1	I have the necessary knowledge to adopt technology in business ventures/initiatives
	KnE2	I have the necessary experience to start/add technology in business ventures/initiatives
	KnE3	I have the necessary technical know-how to start/add technology in business ventures/initiatives
Warm glow (WG)	WG1	Doing eco-friendly business ventures/initiatives gives me a pleasant feeling of personal satisfaction
	WG2	I feel happy contributing to human wellbeing and the quality of the natural environment by involving and initiating eco-friendly business ventures/initiatives
	WG3	By involving or initiating eco-friendly business ventures/initiatives, I feel pleased to do something good for our planet
	WG4	Participating in eco-friendly business ventures/initiatives makes me feel satisfied by giving something back to society and the environment
Green entrepreneurial readiness (GER)	GER1	I have a strong emphasis on R&D, technological leadership, and innovation
	GER2	I have always adopted new technological transformation abilities to create wealth
	GER3	I am always willing to focus all my time and energy on things that impact the business the most
	GER4	I can act fast and seize new opportunities when they arrive
	GER5	I have a strong tendency to be a leader, introducing new products, services, or technology first
Green entrepreneurial intention (INT)	INT1	I will try my best to start and run my green initiative/ventures
	INT2	I decided to establish a company in the future
	INT3	My career goal is to become an entrepreneur

### Sample and Data Collection

The hypothesized model of the study was tested by conducting a digital survey, and the potential respondents were approached by using a social networking application (WeChat). The study collected data from various universities in China in Anhui, Zhejiang, and Jiangsu provinces. The study obtained data from 196 student respondents, encompassing 141 men (71.90%) and 55 women. As far as the variable of age is concerned, overall 160 (81.60%) students were aged between 20 and 25, additionally, 32 students (16.30%) were aged between 26 and 30, and 4 respondents (2.10%) were above 30 years old. The study employed the non-probability sampling technique, where Goddon’s approach was adopted to measure the sample size. Particularly, the study adopted a margin of error of 7% and a confidence interval of 95% for the unknown population size. Specifically, the respondents were contacted at several universities to recruit students through convenience sampling during the first and second quarters of 2021. Additionally, the data collection was a critical process because of the COVID-19 pandemic, therefore data were only collected through the digital medium. The descriptive profile of the collected sample is shown in Table 2. Specifically, individuals who were in the last year of their undergraduate or postgraduate education were taken into account as potential respondents for the current study. Table 2 reflects the demographic variable distribution. As discussed, there were 141 male and 55 female respondents with ages ranging from 20 to above 30. Irrespective of their gender, 97 respondents were graduates, 94 were doing a Master’s degree while 5 claimed they were enrolled in the Ph.D. program.

**TABLE 2:** Descriptive profile of respondents.

Measure	Item	Number	Percentage
Gender	Male	141	71.90%
	Female	55	28.10%
Age	20–25	160	81.60%
	26–30	32	16.30%
	Above 30	4	2.10%
Education	Graduation	97	49.50%
	Masters	94	47.90%
	Ph.D.	5	2.50%

## Analysis

Analysis was performed through ADANCO, SPSS Statistics, and AMOS. The adopted tools helped to address the complexity of our measured model. In the following parts, the internal and external validity were examined, followed by hypotheses testing and moderation analysis.

### Measurement Model

During the internal and external examination of data to measure reliability and validity, confirmatory factor analysis was performed. To compute the internal reliability, the Cronbach’s alpha, factor loadings, and composite reliability were examined which were observed above the lower limit of 0.07 and satisfied the recommended value as advised by Hair et al. (1998). Moreover, the variance extracted was reported for each construct and observed above 0.50, as confirmed by the existing literature (Keren et al., 2021). To test the data reliability, the common method bias test was conducted where the difference by *t*-test was performed to compare the earlier and later half of the collected data. However, no difference between the subsets eliminated the risk of common method bias. Moreover, the single factor test was measured as recommended by Harman (1976). The maximum variance was noted as 37.83% which was lower than 50% of the overall variance. Therefore, the data observed can be reported as internally valid and reliable. The tabular record for internal reliability is reported in Table 3. To further evaluate the concerns related to instrument development, and its reliability, common latent factor testing was performed, where regression analysis of the model with Moon latent factor and without common latent factor were compared. All regression results were measured. However, no difference greater than 0.20 was not observed. Thus, the authors claimed no risk of instrument validity and reliability as suggested by Keren et al. (2021).

**TABLE 3:** Exploring reliability and validity analysis.

Construct	Items	Loadings	α	CR	AVE
Normative support	NS1	0.812	0.753	0.854	0.661
	NS2	0.866			
	NS3	0.755			
Regulative support	RS1	0.739	0.744	0.852	0.659
	RS2	0.865			
	RS3	0.825			
Knowledge and experience	KnE1	0.719	0.792	0.792	0.560
	KnE2	0.773			
	KnE3	0.750			
Market orientation	MO1	0.677	0.738	0.807	0.656
	MO2	0.770			
	MO3	0.841			
Warm glow	WG1	0.814	0.754	0.851	0.587
	WG2	0.794			
	WG3	0.821			
Green entrepreneurial readiness	GER1	0.754	0.756	0.836	0.595
	GER2	0.852			
	GER3	0.700			
Green entrepreneurial intention	INT1	0.792	0.708	0.801	0.629
	INT2	0.782			
	INT3	0.806			

*AVE, average variance extracted.*

To examine the external validity, the correlation scores of each construct were compared with the AVE square root as advised by Fornell and Larcker (1981). Satisfactory results were observed. Moreover, hetero trait-mono trait (HTMT) testing was also conducted as suggested by Henseler (2017). The tabular results are shown in Table 4. All scores in HTMT were lower than 0.85. Therefore, the results confirmed data reliability.

**TABLE 4:** Hetero trait-mono trait scores.

Construct	NS	RS	KnE	WG	MO	INT	GER
NS							
RS	0.243						
KnE	0.425	0.774					
WG	0.529	0.659	0.733				
MO	0.447	0.661	0.605	0.842			
INT	0.722	0.498	0.525	0.892	0.752		
GER	0.351	0.731	0.742	0.684	0.494	0.559	

Apart from the internal and external validity, model fitness was computed as a part of confirmatory factor analysis. Table 5 depicts the fitness of the model as recorded by ADANCO. It shows the values of SRMR, dULS, and dG. These all tests were used to confirm the goodness of model fit. The lowest values show the highest model fit (Henseler et al., 2014). Moreover, the model fitness reported by SPSS-AMOS was reported as NFI = 0.952, CFI = 0.947, and RMSEA = 0.061. The multicollinearity was tested by using SPSS Statistics, where the variance inflation factor of each construct was recorded which was below the upper limit as recommended by Hair et al. (1998).

**TABLE 5:** Estimated goodness of model fit.

	Values	HI95	HI99	Conclusion
SRMR	0.067	0.072	0.075	Supported
dULS	1.067	1.206	1.32	Supported
dG	0.341	0.465	0.505	Supported

### The Proposed Model and Moderation Testing

**FIGURE 1:**
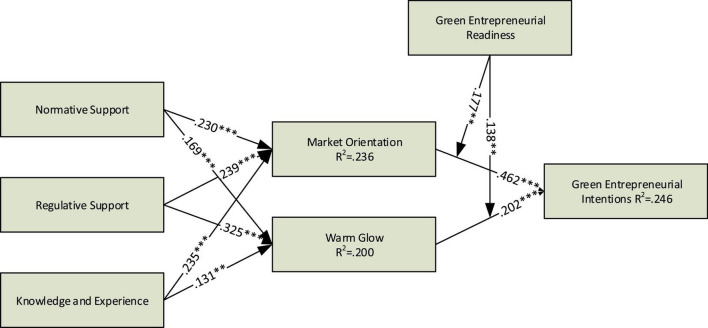
Graphical explanation of the model examined. *** = Significance level of 0.001, ** = Significance level of 0.01.

In the case of the proposed model, the *R*^2^ values reported by market orientation, warm glow, and green entrepreneurial intention were as 0.236, 0.200, and 0.246, respectively. Moreover, the model fitness reported by SPSS-AMOS was NFI = 0.948, CFI = 0.941, and RMSEA = 0.066. In the case of the current proposed model, none of the five proposed hypotheses were reported as non-significant. However, among the causes of marketing orientation and warm glow, regulative support was noted as the most obvious determinant. Moreover, market orientation was also noted as prominent compared to warm glow to define green entrepreneurial intentions. Therefore, it can be concluded that the external environment as a supportive force plays a crucial role in green entrepreneurial intentions, as compared to the intrinsic factor (warm glow). The graphical representation of the results output is shown in Figure 1.

In the case of H5a, the results showed that green entrepreneurial readiness strengthened the positive relationship between market orientation on green entrepreneurial intentions (β = 0.177). The result was computed with the help of hierarchal regression (β = 0.177). Similarly, green entrepreneurial readiness strengthened positive warm glow on the green entrepreneurial intentions (β = 0.138). Table 6 shows the results of moderation analysis.

**TABLE 6:** Hierarchal regression for moderation.

Variables	M1 (β)	M2 (β)	Sig.
**Step-1**			
MO	0.062**		
GER	0.594**		
*R* ^2^	0.265		
**Step-2**			
MO×GER		0.177**	0.03
*R* ^2^		0.283	
Ϫ *R*^2^		0.018	

**Step-1**	**M1 (β)**	**M2 (β)**	

WG	0.297**		
GER	0.043**		
*R* ^2^	0.279		
**Step-2**			
WG×GER		0.138**	0.048
*R* ^2^		0.293	
Ϫ *R*^2^		–0.014	

** = Significance level of 0.01.

## Discussion and Implication

The present study was conducted in the context of green entrepreneur intentions among youth. The study wanted to establish how exogenous factors (normative support, regulatory support, and knowledge experience) emerge and determine GEI. It also presents the unique mediation of warm glow and market orientations to confirm their role to predict green entrepreneurship. Meanwhile, green entrepreneurial readiness operated as the moderator to determine GEI. The study aimed to determine the intentions of youth regarding green entrepreneurship. Along with this, the current venture focused on the regulative support that is helpful for GEI. The findings of the underlined study put forward interesting facts that can be depicted under the umbrella of the research questions in section “Introduction.” Research question 1 (RQ1): To examine institutional factors (normative support, regulatory support, and relevant knowledge experience) responsible for promoting pro-environmental green intentions. RQ1 explained that regulatory support was the strongest exogenous factor (*H* = 0.325) through warm glow to determine green entrepreneurship intentions among youth. Meanwhile, knowledge experience was the least influential factor (*H* = 0.131) to determine intentions to adopt green entrepreneurship.

The results suggested that normative support (H1a) exerted a significant effect on market orientation. However, regulatory support possessed the highest relationship with intentions to adopt green entrepreneurship. Further, the KnE was rated the least significant to map out green entrepreneurship. RQ2 evaluated how the altruistic traits (warm glow, market orientation) trigger the eco-centric green entrepreneurs. In the light of RQ2, warm glow (*R*^2^ = 0.200) had a lower impact while market orientation (*R*^2^ = 0.236) was the strongest predictor in terms of mediation. Consequently, the results depicted that market orientation produced more of an effect to determine green entrepreneurship than warm glow. However, the higher GER increased the association between WG and GEI.

As far as similarities and differences are concerned, the following study has various similarities with previous studies. It posits that normative support indicates green entrepreneurship intentions, and endorses the findings of Ye et al. (2020) who articulated that green entrepreneurship is increased in the context of norms and the collectivist culture. Ye et al. (2020) elucidated that the normative environment is a significant factor to yield entrepreneurial activities in society. On the contrary, Hussain et al. (2021) anticipated that subjective norms have no association with green entrepreneurship. Chen et al. (2018) put forward that knowledge has the key factor to influence green entrepreneurs’ activities which is similar to the present study model. Hartmann et al. (2017) anticipated that warm glow would be the most significant driver for promoting green behavior.

### Implication

The present research suggests various theoretical and practical implications. It is the novelty of the study that institutional theory has been employed for the first time to seek out green entrepreneurial intentions. In the present study, normative support, related knowledge, regulatory support, and experience were used as uncontrolled variables. And market orientation and warm glow were the mediating variables of this research. As far as the unique aspect of the study is concerned, green entrepreneurial readiness was employed for the first time as a moderator in this model. In addition to this, this study contributed by taking warm glow as an altruistic trait as a mediator. Based on the findings, the study unveiled that the majority of youth (42.8%) were inclined toward green entrepreneurship. The significant role of regulatory support accelerated green entrepreneurship.

The practical implications of the study are also unique contributions in this regard. The study examined the impact of the institutional factors on market orientations. Similarly, the study also confirmed the relationship between all these indicators and warm glow (altruistic trait). The study implied that institutional forces had never been discussed earlier with green entrepreneurship. The current research provided leads to future planning at the national level. A total of 37.8% of the respondents believed that regulatory support assisted entrepreneurial activities. In total, 23.5% agreed that political regimes, either local or national, supported entrepreneurship. Similarly, the study puts forward the findings that a notable number (36.7%) of respondents claimed that the government supported innovative ideas. However, the insertions must increase their efficiency if they want to execute entrepreneurial ventures. It will address the issue of unemployment and environmental degradation. The respondents of the study were university students; however, there are several constraints in this study. The first limitation of the study is that it only investigated students rather than seeking out the overall population. Future studies can examine this model in low-income earners and job holders as many of them are aspirants of green entrepreneurship. However, this study employed warm glow and market orientation as mediators. In future studies, the mediators can be changed from the consumption theory by choosing any two factors. The overall factors that constitute the consuming theory are conditional value, functional value, epistemic value, social value, and emotional value. Two factors can be extracted as a mediator from the respective theory. Similarly, instead of behavioral modeling, qualitative research can be executed to comprehend the green entrepreneur intentions among youth. Green entrepreneurship is replacing the traditional market industry and it has a bright future ahead owing to solving various socio-economic issues. The promotion of the altruistic factor can surge green entrepreneurship by making individuals responsible for society. Green entrepreneurship must be included in the curriculum to increase motivation. Hence the students may join green entrepreneurship. Therefore, the production segment can also be responsible by fulfilling social corporate responsibility.

### Future Studies

The present venture can be further stretched by comparing the opinion of youth on green entrepreneurship intentions in any other region across the globe. Moreover, the increasing climate change concerns and environmental awareness can be sought out as a variable to map out green entrepreneurship intentions. In addition to this, the findings of the study can be re-assessed by changing the other models that help to adopt pro-environmental behavior. Green entrepreneurship intentions can be probed through the qualitative methods of investigation as well. To address instrument concerns related to adapted instruments, a qualitative study can also be conducted.

## Conclusion

Environmental problems can be addressed by green entrepreneurship intentions. The rationale behind this reality is that entrepreneurs are creative, have modern skills, and provide alternatives to resources that can be good for environmental advantages. In this research, the authors have investigated the mechanisms of the external environment that spur green entrepreneurship intentions; in this venture, the researchers have explored the readiness and opportunity recognition that contribute toward green entrepreneurship through theoretical and practical implications. The investigated study can confirm that normative support, related knowledge, regulative support, and experience can advance green intentions by recognizing the opportunity. This study has fulfilled the gap persisting in the academic circle to determine the intention toward green entrepreneurship. The study concluded that the role of governmental policies and laws were the most influential aspect of intentions to adopt green entrepreneurship. As it is evident that governments can have a more effective role to persuade the masses toward GEI than normative norms. The rationale behind this phenomenon can be the role that people play and the laws they follow rather than their norms or social norms. This also points toward the limitations of the study because green entrepreneurship can be further studied in terms of any other theoretical basis.

## Data Availability Statement

The raw data supporting the conclusions of this article will be made available by the authors, without undue reservation.

## Ethics Statement

The studies involving human participants were reviewed and approved by the Jilin University, China and Bahauddin Zakariya University, Pakistan. The patients/participants provided their written informed consent to participate in this study.

## Author Contributions

YW: conceptualization and lead. AP: analysis and methodology. MF: initial draft. MM: data collection. SH: proofreading and revising draft. All authors contributed to the article and approved the submitted version.

## Conflict of Interest

The authors declare that the research was conducted in the absence of any commercial or financial relationships that could be construed as a potential conflict of interest.

## Publisher’s Note

All claims expressed in this article are solely those of the authors and do not necessarily represent those of their affiliated organizations, or those of the publisher, the editors and the reviewers. Any product that may be evaluated in this article, or claim that may be made by its manufacturer, is not guaranteed or endorsed by the publisher.
